# The Hospital. Nursing Section

**Published:** 1903-04-11

**Authors:** 


### The Hospital.

### Nursing Section.

Contributions for this Section of "The Hospital" should be addressed to the Editor, "The Hospital" Nursing Section, 28 & 29 Southampton Street, Strand, London, W.C.

No. 863.—Vol. XXXIV. *SATURDAY, APRIL* 11, 1903.

### Notes on News from the Nursing World.

### THE LATE DR. SOLOMON SMITH AND THE WORKHOUSE NURSING ASSOCIATION.

By the deeply-lamented death of Dr. Solomon C. Smith, the Workhouse Nursing Association, whose report for 1902 has just reached us, has lost one of the members of its Executive Committee. Dr. Smith always took the greatest personal interest in all movements affecting the welfare of nurses, and was a strenuous advocate of every effort put forward for the elevation of the various branches of the profession. He made a special study of the question of nursing in workhouse infirmaries; and we are sure that his unfailing sagacity, his admirable judgment, and his ripe experience were as warmly appreciated by his colleagues on the committee of the Workhouse Nursing Association as they were by all who were brought closely into contact with him, and had the opportunity of appraising the character of a man with whom it was a privilege to work.

### THE CLOSING CEREMONY OF THE WOMEN'S MEMORIAL TO QUEEN VICTORIA.

Nothing has yet been definitely settled respecting the closing ceremony of the Women's Memorial to Queen Victoria, and will not be until the King and Queen are both in England together. As we have already intimated, it is probable that so far as the sum collected by the Central Committee is concerned, Lady Londonderry, at a private gathering, will hand their Majesties a cheque for the amount collected. Possibly, Lady Cadogan will at the same time make a presentation of the sum obtained in Ireland; and then, or subsequently, the Duchess of Buccleuch will hand over the contribution of Scotland. But no definite arrangement has yet been made.

### ROYAL VISIT TO KINGSTON-ON-THAMES.

It is characteristic of members of our Royal Family that, from the Queen downwards, they are always ready to undertake any function within their power for the benefit of the sick and suffering. The Duchess of Albany, after an absence of more than three years, will return to Claremont at the end of this month, and she has graciously consented to open a new home which has been acquired for the extension of the excellent work of the Kingston-on-Thames Nursing Association. The date is not yet fixed, but it is expected that her Royal Highness will find it convenient to perform the ceremony at the end of next month, or early in June.

### THE CHANGES IN THE INDIAN ARMY NURSES.

Several more or less inaccurate statements have been made respecting the Indian Nursing Service, but we are now in a position to give the facts of the case. In accordance with the unanimous wishes of the members of the Service, it is not to be absorbed in the Imperial Military Nursing Service, but is to remain an independent organisation. The control will, therefore, still be vested in the India Office, and the Secretary of State will approve the appointments made. But the selection of members of the Service will, in future, practically rest with the ladies who will act in an advisory capacity. One of these, Miss Mary Adelaide Herbert, lately matron of Worcester General Infirmary, who was trained at St. Thomas's Hospital and was subsequently sister, night sister, and assistant matron, has already entered upon her duties at the India Office. The other lady is Miss Catherine Grace Loch, senior lady superintendent of the Indian Nursing Service, who is at present on leave in England. Miss Loch was trained at the Royal Hants County Infirmary and St. Bartholomew's Hospital, where she was afterwards sister. She has been a member of the Indian Nursing Service since 1888, and her last appointment was that of lady superintendent at the Station Hospital, Rawal Pindi, Punjab. Miss Loch has won two great distinctions, the decoration of the Royal Red Cross, and the Indian medal with "Hazara 1888" clasp. The Service is to be strengthened in the course of the summer by the appointment of 16 additional sisters, and it is in contemplation to further increase the staff a little later on. The 16 selected candidates will proceed to India in October, and funds have been set aside for the provision of suitable quarters for them. It has not yet been decided where they will be located.

### NURSING BY NUNS.

In the House of Commons last week a motion was agreed to, at the instance of Mr. Sloan, for a return showing the number of workhouse infirmaries in Ireland in which nuns are employed in any capacity, the number of nuns so employed in each infirmary, and the amount paid to them by way of salaries within the last financial year. The inquiry now proceeding at the Granard Workhouse will invest the return with particular interest.

### NIGHT NURSING BY PAUPERS AT COCKERMOUTH.

The Cockermouth Board of Guardians have brought down upon themselves a strong and well-deserved rebuke from Mr. H. Jenner-Fust, the Local Government Board Inspector. At the last meeting Mr. Jenner-Fust pointed out that night nursing in the infirmary was entrusted to paupers, who fell asleep and neglected their patients. So far, he said, no steps had been taken to remedy the defect in the nursing staff, although there were 68 patients in the sick ward and only one nurse to attend to the whole of them day and night. In this respect Cockermouth Workhouse stood alone, and in order to bring themselves up to the level with the rest of the district the guardians should have a superintendent and four nurses. Mr. Jenner-Fust observed that he was quite aware that the Cockermouth guardians hold the Orders of the Local Government Board cheaply, and that they carried them out or not at their own sweet will, but, he added that, if other unions did the same it would be necessary for the Local Government Board to apply to Parliament for more extensive powers than they at present possess. There is one comment to make on this disgraceful state of affairs. Mr. Jenner-Fust having assured the guardians that pauper nursing is absolutely illegal at the present time, if it be not at once abandoned at Cockermouth the board should be prosecuted.

### NURSES OF EIGHTEEN.

It will, we hope, be observed by the President of the Local Government Board that even the Executive Council of the Poor Law Unions' Association do not approve the recommendation of the Departmental Committee on Workhouse Nursing to create a new order of "qualified nurses." These gentlemen, 24 in number, met last week in London in order to discuss the report of their own committee on the Departmental Report, and came to the conclusion that the title of the second grade of nurses should be simply "nurse," instead of "qualified nurse." One speaker was not satisfied with the alte ation, and said that a term was required to show that certain nurses are only partially trained. The title "nurse," for a person with one year's training, is preferable to that of "qualified nurse," but it is misleading. Moreover, the Poor Law Unions' Association propose that the title of "charge nurse" should be substituted for "trained nurse," which is certainly not an improvement on the report of the Departmental Committee. In spite of strong objections on the part of three of the Council, the majority recommended that the minimum age for appointment as probationer should be 18 instead of 21. This, we can only suppose, will be disregarded. Young persons of 18 are not old enough to undertake the duties of nursing adults.

### DRIVES FOR NURSES.

It has often been suggested in these columns that one of the favours highly appreciated by tired nurses is the opportunity of enjoying a drive in the country, and we notice with interest that a nurse and three patients at the London Hospital have had a jaunt in a motor car. The owner of the car did not wish his or her name to be known, and it appears that when the driver arrived at the hospital, he said he was instructed to take "some nurses and patients out for a ride," but not to give the name of his employer. All's well that ends well, and the party returned in safety, highly gratified with their experience of motoring. But, however kind-hearted people may shrink from having their good deeds published abroad, we think that on any future occasion when a drive in a motor car is offered, the driver should be allowed to disclose the identity of his employer in confidence to the hospital authorities, who are responsible for the security of both patients and nurses.

### DANGEROUS EASTER EGGS.

In view of the number of Easter eggs likely to be distributed this week, the following facts are particularly suggestive :—Recently a nurse received a telegram to go to two children of three and five, supposed to be sickening for measles. They had had a slight rash which had, however, died away. Their temperatures varied from 99° to 102°, and they both suffered from sickness and diarrhœa. They had lost flesh to a serious degree, and had been ill already nearly a week. The doctor was thoroughly puzzled, as the symptoms of measles did not develop, but incidentally the nursemaid told him that a few days previous to their illness they had been to a small party, and had been given chocolate eggs. They ate the outside, but gave a small white tablet, found in the centre, to the nursemaid, saying it was nasty. The nursemaid said that "Poison" was written on these tablets, and the words "Light this and a serpent will appear." The chocolate had evidently become impregnated with the phosphorus, hence the illness. It was ten days before the children were downstairs again, and some weeks before they "put on flesh."

### NURSING STAFFS IN WORKHOUSES.

A return which has been compiled by Mr. E. B. Wethered, Local Government Board Inspector for a portion of the West of England, comprising Gloucestershire, Hampshire, Somersetshire, Staffordshire, Wiltshire, and Worcestershire, shows that while the number of patients in the workhouse infirmaries in his district rose from 2,925 in January 1899 to 3,244 in January 1903, the number of patients to each nurse decreased from 17 to 14. Imbeciles and epileptics in special wards dropped from 1,111 to 1,081, and the number in charge of each nurse from 19 to 16. The number of pauper attendants has dropped from 312 to 195, of whom 72 were employed last year in Worcestershire. The joint inspectors for Lancashire, Westmorland, and part of Cumberland, Mr. H. Jenner-Fust and Mr. W. M. Moorsom, report that on January 1st, 1903, 10,242 patients were in the infirmaries, that 605 nurses were on day duty, and 204 on night duty. The increase of nurses is 33, and while the number of patients to each nurse the previous year was 12·73, it is now 12·66. This is a slight improvement, and we learn with satisfaction that "the number of paupers employed under the name of attendants continues to decrease." There is still "great need in several instances for more night nurses."

### LONDON SCHOOL NURSES' SOCIETY.

An appeal has been issued on behalf of that excellent organisation, the London School Nurses' Society, which exists for the purpose of supplying visiting nurses to the poorer public elementary schools. The Board of Education, in an official publication, has expressed high approval of the purpose in view, and speaks of "the value of these nurses in improving the health of the children and securing regularity of attendance." There are, however, nearly a thousand public elementary schools in London, and the Society has only sufficient money at its disposal to provide 75 nurses. Obviously, a great many more are needed if the work is to be thoroughly done. Subscriptions should be sent to the honorary treasurer, Miss S. Lawrence, 44 Westbourne Terrace, W.

### ISLINGTON INFIRMARY NURSES.

Fifty probationers of St. Mary's Infirmary, Highgate Hill, were last week presented with bronze medallions by the Guardians of the Islington Union. The medallions were awarded to those nurses who, having completed at least one year of their training, had also passed the prescribed examination, and had given satisfaction in respect to the punctuality with which they had attended to their duties, obedience to the rules, tact and kindness in the management of patients, personal neatness, and general intelligence. The presentation was made in the Board Room by the Chairman of the Infirmary Visiting Committee, in the presence of several of the Guardians, Mr. S. Lambert, Chairman of the North-Western Fever Hospital, the matron, Miss Alice Little, and the assistant matron, Miss Eyles. Mrs. Marshall and other Guardians made a few remarks appropriate to the occasion, and the medical superintendent, Dr. A. H. Robinson, said that the all-round results of the nurses' examination were very satisfactory. Owing to the difficulty of getting fully-trained nurses, it had been decided to train the entire staff in the infirmary, and to grant a three-years' certificate. Dr. Robinson urged that nurses who had finished their three years' training ought to consider themselves still as "fledglings," and he would advise them when certificated to go to some hospital where they would gain fresh experience. A doctor considered himself still on the threshold when qualified, and a nurse should do the same. The medallions represent the Virgin and Child, with the motto, "Amor et spes omnia vincunt," surrounded by a scroll bearing the words, "Saint Mary's Islington Infirmary." They are to be worn with indoor and outdoor uniform. It is probable that a gold medallion will be given to the nurse taking the highest place in the final examination.

### BIRMINGHAM NURSES AND THE NATIONAL PENSION FUND.

At the annual meeting of the Birmingham and Midland Counties Institution for Trained Nurses it was stated that the establishment of a benevolent fund for the relief of nurses during prolonged illness, and the arrangement made in connection with the Royal National Pension Fund for Nurses, would entail an extra annual expenditure of £550. But, happily, the institution can afford to bear this very wise outlay, and probably, because of it, will become increasingly prosperous. Last year, after £535 had been allotted by way of percentages to the nurses, there remained a balance on the revenue account of £688, in addition to which a legacy of £436 had been received. The number of cases attended was 721, and there are now 88 nurses on the staff. A special gift of £50 has been made, at the instance of the committee, to the District Nursing Society, besides the quarterly contributions which in 1902 came to £170. We are glad to learn that the popular lady superintendent, Miss Ewing, is recovering from her serious illness.

### AWARD OF MEDALS.

Miss S. Fussell who has been appointed superintendent nurse at Keynsham Workhouse Infirmary, near Bristol, has, on the completion of her three years' service of the Northern Workhouse Nursing Association at Rotherham Infirmary, been awarded the silver medal, which was presented to her by the Chairman of the Rotherham Board of Guardians. Miss Williams has also received the silver medal of the Association, and in her case the little ceremony of presentation was performed by Miss Siddon, Vice-Chairman of the Huddersfield Board of Guardians at the last meeting of the Visiting Committee.

### FOOTBALL AND NURSING.

The closing days of the football season are happily marked by contributions on the part of several of the clubs to the funds of nursing organisations. Thus, the Reading Football Club has voted the sum of £21 5s. as their share of the Southern Professional Charity Cup Competition to the Victoria Nursing Home in the town; and the Woolwich Early Closing Football Club has handed over to the Woolwich, Plumstead, and Charlton Nursing Association the sum of £5 1s. 1d., the result of a match on the Manor ground last month. In both cases letters of warm appreciation on the part of the Nursing Associations have been received by the secretaries of the respective clubs.

### THE DEARTH OF POOR LAW NURSES.

A striking illustration of the dearth of workhouse nurses is shown by the experience of Totnes Guardians. They have been seeking an assistant nurse for some time, and received an application from a nurse who was formerly at Chippenham. They gladly appointed her, but last Saturday they had the disappointment of receiving a letter from her offering apologies for not being able to take up the position. She had applied to several Boards of Guardians in the hope of being among the selected candidates somewhere but found herself appointed to three places. Totnes Guardians are still waiting for another application.

### GRATEFUL GUARDIANS.

The Clerk to the Guardians of the Battle Union has written to the Hon. Sec. of the Meath Workhouse Nursing Association as follows: "I am instructed by the Guardians of this Union to ask you to kindly convey to Lady Meath, the President of your Association, their grateful thanks for the great help rendered by the Association at various times in providing nurses for the Infirmary at the Workhouse of this Union, when the Guardians had experienced the utmost difficulty in obtaining such officers, notwithstanding repeated advertisements, at considerable expense."

### SHORT ITEMS.

The new Nurses' Home at the Middlesex Hospital is practically finished, and several of the bedrooms are already occupied. Thirty nurses are to be added to the staff.—It has been decided to form a District Nursing Association at Knaresborough, a nucleus being contributed by the balance of £17 of the Coronation Festivities, and £40 from the Diamond Jubilee Fund.—The s.s. *Briton*, which arrived at Southampton on Saturday, had on board Nursing Sisters E. A. Deacon, and N. Woodman, M. Glenn and H. Luart of the Army Nursing Service Reserve.—The s.s. *Nubia* arrived at Southampton from South Africa on Monday, having on board Nursing Sisters M. Little, M. Moore, A. L. Stuart, and E. E. Thomas of the Army Nursing Service Reserve.

### The Nursing Outlook.

"From magnanimity, all fear above;
From nobler recompense, above applause,
Which owes to man's short outlook all its charm."

### THE MIDWIFE OF THE FUTURE.

Last week witnessed the official birth of the Central Midwives' Board. It is an event of the highest importance, and the developments of the next few months will be watched with the keenest interest. Probably no board, of corresponding kind, ever constituted has had responsibilities placed upon it of so wide and far-reaching a character. The Midwifery Bill of 1902, as it was passed, touches the veriest fringe of the subject it deals with, and looking through it again as it lies before us, it seems strange to remember how hard every line of it was fought. Nominally it settles everything, practically it settles nothing. No one ever objected to midwives in principle, and the only real question was what should a midwife be? Midwives indeed have existed since the Year One, and under this Act there are to be midwives still. It provides that they shall be "registered" on production of some variety of certificate, but it no more determines the practical meaning of midwife or on what lines she shall be manufactured than the Pedlars Act settles what a pedlar shall peddle, or how he should sell his wares. With one exception; a pedlar must not sell spirits and must keep himself reasonably sober, and a "registered midwife" must eventually have some sort of education, and must not attend cases "of abnormality." But what sort o education, and what are cases of abnormality? These and all other really controversial questions connected with the subject are left to be settled by the Central Midwives' Board. Hence the immense importance of its constitution, and of the decisions at which it has to arrive. The task before it is one of immense difficulty, and its decisions to be satisfactory must prove deep knowledge on the part of its members of all the points at issue, and be of statesmanlike wisdom.

And this is just where we quarrel with the Act. It has always been a function of councils under acts to draft regulations and see to their observance, but hitherto it has been no part of their duty to determine great questions of public policy, and this is exactly what the Central Midwives' Board has to do. Shall the midwife of the future be a decorated but only slightly more educated edition of her predecessor, or shall she be a woman so educated as to think herself quite capable of competing with the medical practitioner, so far as, while doing so, she can manage to keep within the letter of the law? This is the question which has now divided midwifery reformers into two opposing camps, and these are the views they respectively hold, though they might object to the words in which they are described.

Curiously enough the keenest enthusiasts of the midwifery movement at its inception, now seem almost frightened at the result achieved. To the first party these belong, and their cry is cheapness. "Give us," they say, "an educated midwife if you can, but in any case give us a cheap one, a woman of the people who will live with the people, and do everything that the [people or anybody else can wish, but cost neither the people nor anybody else anything." The other party does not speak quite so loudly, but is equally determined, and if either of them gets its way, every object which reformers originally had at heart will be defeated, and the last state of the lying-in woman of the poorer classes will be as bad as the first. Women registered under the first scheme will be practically little more educated than those they are designed to replace, though much more self confident; and those under the more ambitious scheme will cause constant friction with the medical profession, of which the lying-in woman will be the last to hear, but the first to suffer from.

How the board will steer between these two parties remains to be seen, but there is one point which it is to be hoped they will keep clearly in view from the first. Cheapness does not come into the question. Associations will arise and, indeed, are already being formed, which in the poorest districts will make the midwife independent of the direct fees of her patients, and in the others they will be able to look after themselves.

The ideal line, if any, would be for the Board to develop the movement which had already commenced before the reformers had their say, viz., the taking up of midwifery by fully-trained three-years nurses. They by their training have learned discipline, have learned to respect superior knowledge, learned to call for its assistance when needed. Thus, and perhaps thus only, will the true purpose of the Act be fulfilled, and perhaps even something more. For no woman who has been a three-years nurse can quite resist when brought in contact with "a case," and thus will the lying-in woman receive some of that skilled after-attendance which she requires just as much as help in the bitterest hours of her need. This is a point indeed which midwifery reformers never seem to have grasped. They have noted the evils of existing conditions without stopping to consider their causation. Ignorant or careless or meddlesome midwifery is one source of evil, but the absence of any provision for skilled after-care is another at least as great. There are very few places where even the poorest women cannot already obtain skilled attendance if they seek it, and unless the new creation is a skilled nurse as well as a skilled midwife the Act will fail in its purpose.

### Lectures on Medical and Surgical Nursing.

By John Hopkins, F.R.C.S., Medical Superintendent of the Central London Sick Asylum District Asylums, Cleveland Street, W., and Hendon, N.W.

### LECTURE V.—TOXIC DISEASES.

When a person has had an attack of fever he loses his susceptibility to it. His chances of taking the fever again are slight. That is the rule, though there are some few people who take the same fever several times. The reason of this has been discovered by experiment upon animals. Microbes have been injected into their tissues until it is found that they have no further effect. The animals have become indifferent to their presence. When the serum from these animals is taken it is found to destroy the microbes when placed in it, and when it is injected into the tissues of another animal it is found that the microbes will not grow in its tissues either. This discovery has been made use of for the prevention and cure of some diseases both in animals and in man. You know already that microbes in the tissues give off a substance that destroys white blood cells, and that these blood corpuscles in their turn throw off a substance that kills the microbes. These two powerful agents are called toxins and antitoxins. The white blood cells secrete an antitoxin which neutralises the toxin and renders it incapable of doing harm. This secretion of antitoxin by the blood cells does not cease with the disappearance of the microbes. A new habit is set up in their mode of life, which lasts for days only in some cases, but in others for years even, so that the blood is kept supplied with a quantity of antitoxin sufficient to prevent a person from taking the same disease again for a certain period.

*Diphtheria* is one of the diseases treated by the injection of antidiphtheritic serum hypodermically. It is a disease characterised by the growth of a microbe in the throat, that causes the excretion of a coagulable fluid from the mucous membrane where it rests and grows. This excretion is not like patches of mucous that are easily scraped off, but is firmly fixed to the mucous membrane, and when forcibly detached is found to peel off like a bit of rind. This is called the diphtheritic membrane, and it spreads, getting daily larger and larger, till it covers the back of the throat, the palate and fauces, and spreads down into the larynx, which it often blocks up in infants, and leaves such a narrow channel for the breath that the trachea has to be opened to prevent suffocation. The toxins of diphtheria getting into the blood cause albuminuria, and paralysis of various parts, the blood-cells being unable to produce antitoxin in some people sufficient to neutralise the toxin of the diphtheria. The nursing of the disease is of the greatest importance. The throat requires special attention, and a nurse needs to have gained considerable skill to be able to spray, syringe or swab a throat effectually, especially in young children. No opportunity should be lost in acquiring all possible practical skill in this. When the larynx is affected, and laryngeal spasm is imminent, watchful care is needed lest the patient die before help is called in time; for at any moment it may be necessary to perform tracheotomy. For this the nurse should have everything in readiness and at hand. After the operation the air of the tent-bed—in which the case should be put—needs to be both warm and moist, and the tube in the larynx requires to be kept clear and free from mucus; for it is apt to become blocked, the result of which unless promptly attended to is asphyxiation.

*Tetanus* generally follows upon a wound, though this may have quite healed before the presence of the tetanus microbe declares itself. As a rule, there is a suppurating wound present somewhere in the body when the symptoms begin. The earliest symptoms are in connection with the wound, but generally pass unnoticed. It is only when the bacillus, which grows in the tissues about the wound, has given off toxins, which have been absorbed into the blood, that the symptoms are taken notice of. These are peculiar movements due to muscular spasm, and pain due to the same cause. Stiffness of the neck and muscular twitching, especially about the face, are generally first noticed. Presently the spasms become more violent, and affect various sets of muscles. Death is a common result, but patients sometimes recover, though slowly. In the nursing of such a case, it is to be remembered that anything likely to excite a spasm is to be avoided, and as the whole nervous system is highly oversensitive, the least noise, a bright colour, the presence of fluid in the throat, and touch even may excite a spasm; so that a subdued light, quietude and rest undisturbed as far as possible are indicated. By thus avoiding spasm, the strength of the patient is preserved until the poison has worn itself out, or has been neutralised by treatment, with antitetanus serum. In feeding the patient, this must be done as seldom as possible, and as this causes spasm, it is best given by stomach tube or as enema whilst the patient is under an anæsthetic.

*Hydrophobia* is a form of muscular spasm similarly excited by very slight stimulation as in tetanus. An attempt to swallow water brings on a violent spasm of the muscles of the throat and the fluid is ejected. Presently the sight of water or hearing it poured out, is sufficient to cause the spasm, so readily apparently are the channels of association opened up. Hence is derived the name, hydrophobia, which means fear of water.

When once the disease has betrayed its presence, there is little or no hope of recovery. But the prevention of it after the bite of a rabid dog has been proved possible at the Pasteur Institute in Paris, to which people are sent that have been bitten.

*Snake Bite* is often fatal, especially in warm climates. The snake poison when introduced into the tissues excites the white blood cells to form an anti-venom. By this means an antidote to snake poisoning has been produced which will neutralise the venom if injected immediately after the bite has been inflicted. The snake charmers of the East are said to render themselves immune to snake poison by allowing young snakes, while as yet their venom is weak, to bite them from time to time.

### TRAVEL NOTES AND QUERIES.

Spring at the Italian Lakes (Nanfans).—I do not keep copies of my letters, so that I do not know what I recommended you, but most decidedly do not attempt too much. I think the amount you speak of is rather little for the Italian lakes, as the journey is expensive. The Loire would be very nice. Articles on it came out in The Hospital on May 12th, May 26th, and June 9th, 1900, they will give you plenty of information. It would not be too warm in Italy in May. I should recommend you to take entirely woollen clothes, thicker and thinner; the sun is very hot and the shade strikes cold by contrast. Have two tailor-made dresses of wool of different degrees of warmth, it will be all you need. Take a golf cloak, not too heavy, and a dust cloak in preference to a waterproof, if both cannot be had. Write me again if I have left anything unexplained.

Cromer Rest Home for Nurses (A.B.).—Your letter and prospectus will be most valuable and I will gladly recommend it.

### Words of Advice to Nurses.

BY ESTHER H. YOUNG.

### LECTURE IV.

(*Continued from page* 224.)

How very foolish some nurses are about dress! they waste a great deal of money and time on extravagant and most unsuitable garments; never forget that you are nurses, servants of the sick, and do please be neat always, away from hospital as well as in it. Manly costumes are out of place; tails to your skirts are untidy and collect dirt; don't be tadpoles, with long frivolous tails! any extremes of fashion are unbecoming. Nurses in ordinary dress cannot be too quietly or neatly attired. Cultivate the habits of neatness and quietness while you are in hospital, and then it will always be natural to you in your work and uniform.

Then, again, the way in which a nurse spends her holidays has much to do with the education and training of the mind. When away from work enjoy yourselves as much as possible, but in a womanly way, and in a way befitting your calling. Most of us have read Lord Roberts' letter to us at home about his soldiers' return—apply this sentence to yourselves —"They bore themselves like heroes on the battlefield and like gentlemen on all other occasions." Bear yourselves like nurses on duty and like gentlewomen on all other occasions. So be careful how you dress; don't suppose that this "does not matter," it does matter very much indeed!

As in dress, so in other ways. Don't get callous about your self-respect, or possibly almost lose it. Be careful in your respect for fellow workers, students, and patients. It hurts me to hear the way some people talk about nurses, yet I am afraid they have reason for so doing. "I would never let a sister of mine be a nurse." "Oh! no, I would rather let my daughter do anything than go into one of those dreadful hospitals." Why, oh! why are such things said? Is it not because we are not careful in our behaviour, our dress, our customs, our manners? And is it not because in dealing with the students with whom we work we are often so unduly familiar, so lacking in self-respect, joking on subjects that we should not, gossiping over the patients' beds? Can we not show them all due politeness, help them all we can, work with them, together do our best for the patients on whom we are both learning, and behave like grown men and women—students of a particular branch of learning, and not like schoolgirls and boys?

Remember, nurses, much depends on yourselves. The students will not, cannot make you flirt, or behave as you should not, unless you plainly show them that you wish it. If you just quietly let them see that such conduct in hospital is unseemly, it will cease, there will be no more difficulty about the matter. As I said before, go out as much as possible, go to friends, go home when off duty; but play while you play, work while you work.

Again it hurts me most acutely when I hear people objecting to the life of a nurse, because of "the things that she does and sees." I heard of a probationer entering a hospital one day and leaving it the next, because she was sent to the men's ward; she had no idea she would see men in bed! A fond mother went to see a matron before bringing her daughter, the would-be candidate, and said must her daughter do things for men patients? Might she at least begin with women? These notions about the work of a nurse are due, I am afraid, to lack of self-respect and respect for others in some of our sister nurses in the past. A real nurse should be able to do anything for a sick man, woman, or child, without a thought as to niceness as to sex; in fact, with only the one thought in her mind, of alleviating suffering, of caring for her patient. She should so absolutely forget herself as not to notice, not be aware of, nor let her patient for one instant feel that there is anything unnatural in the necessary attention, whatever it may be. Here I would especially say a few words to those who are going out private nursing. You will have to do so much more for your patient, and he or she will dislike it so much more than your hospital patient. You will not have the help of the students, who, in hospital, do so many things for the men. And your woman patients will be so much more sensitive than the woman you nurse in your ward.

At first they will even dislike being washed by you. If they are able let them do what they wish for themselves. A man hates to have his face washed by a woman. Nurses carry far too much hospital red-tape into private houses; they take possession of the lady or gentleman, the master or mistress of a house perhaps—and show them so little respect that one need not wonder why they sometimes dread the advent of a private nurse. Always wash your patient under a blanket, yet always know that the patient is absolutely clean. I remember going to help nurse a clergyman, suddenly taken in the street with paralysis—probably all his life he had been scrupulously clean. He had been ill a few days, and from his waist was helpless. I was on night duty, and he was in a semi-conscious state, but in the morning, as a matter of course, I washed him thoroughly, and with due and proper care and respect as I had been taught to wash helpless patients—men and women. I shall never forget his unspoken but evident gratitude. His day nurse, thinking of herself and her "un-nurse like" feelings, and lacking the respect and reverence due to that helpless body, had let it suffer from want of cleanliness. It is when we leave these things undone, or do them without the right feeling, and without respect to the patient's feelings that we bring discredit on the details of our work. In this—particularly, let our best side come to the front; no unpleasant bits of our work must ever let us become callous or hardened but rather the reverse; our better selves (and we all have a better self) should give a good tone to the whole of our work.

I have put before you the need of mental cultivation and education for yourselves, and I also presumed to touch upon the need of care respecting your own souls: bear with me for a few minutes if I once more touch upon religious subjects and remind you that your patient has a soul. I don't ask you to continually hurl texts at him or ask what to him appear to be impertinent questions; but, in hospital or in private nursing, don't forget to pray for him, and don't be too shy to ask him if he would like to see the parson. In hospital, at the ward services and prayers, be as quiet and reverent as possible, even though you may have to go on with your work. Example is better than precept. Never make jokes about any religious efforts made in the ward in which you work. Teach them to treat with respect clergy, lay readers, and ladies with flowers and texts, etc., etc. Any attempt to reach them is done with the best intention and from the highest motive, and will do some good, though possibly not according to your views. Help the little ones to say their prayers, help all—men, women and children—as you best can and as opportunity occurs. Then about the minds of your patients: you can find games and books for the men, get the men to tell you the news from their papers; give the women work, and the children toys.

(*To be concluded*)

### Everybody's Opinion.

[Correspondence on all subjects is invited, but we cannot in any way be responsible for the opinions expressed by our correspondents. No communication can be entertained if the name and address of the correspondent are not given as a guarantee of good faith, but not necessarily for publication. All correspondents should write on one side of the paper only.]

### DORSET COUNTY HOME FOR NURSES.

"The Honorary Secretary of the Dorset County Home for Nurses" writes: As you were kind enough to comment in your last issue in appreciative terms of this home, I think it is due, both to your readers and to the friends of the home itself, that I should state that as far as I know there is not the slightest intention of adopting any "retrograde suggestion" as to the employment of nurses "of a lower qualification than the existing staff possess." In their annual report to the subscribers, the committee distinctly state that, although they are anxious to enlarge the staff, they are determined to engage none but thoroughly-qualified nurses; or, to use your own words, to maintain "the reputation it has won for supplying only first-class nurses." The proposal made at the annual meeting had no reference to the nurses on the staff, it merely aimed at assisting those country villages which required resident district nurses, by training for them free of expense such local women as might be useful for maternity work, and for the lesser complaints of daily life. It was considered that in this way a new branch of work might be undertaken, which would assist the poor in their homes, while the county institution would still retain its staff of fully-trained, first-class nurses to carry on the work which is so justly appreciated.

### ADVICE TO NURSES.

"M. Holt-Houghton" writes from Guthrie Lodge, Albert Road, Clifton: I note with pleasure the articles recently published by Miss Young in the *Nursing Section* of The Hospital, and also the excellent remarks on "Character" in the article on "Higher Education." While all matrons of standing desire that their nurses should be of high moral character, do they do all that might be done to procure and keep young women who have the elements of such a character? In one of London's very finest hospitals it was a constant matter for regret that those who were the most refined and cultivated were the first to break down under the strain of work, the coarser-fibred as a rule reaching easily the end of their training. As an experienced sister expressed it, "If a girl thinks and feels she soon wears out." Yet the ideal nurse should "think and feel." Could not this difficulty be met by using a few more wardmaids, and fewer nurses but choicer ones for the actual duties of nursing? Again, do matrons make all the inquiries they might about previous character? I ask this after meeting in a tram last week a servant, discharged for a serious theft, in the uniform of a large local hospital.

### CROWN COLONY SALARIES.

"An Occasional Correspondent" writes from the Far East: Your correspondent of December 27th does not yet seem to grasp the fact the subject under discussion was not the advantage or disadvantage of a sterling currency to future nurses, but the unfairness to those now under argreements who are not offered an equivalent of their present salaries. As to their benefiting by purchasing food at dollar rates when receiving sterling payments, may I point out, first, that Hong Kong literally has no produce of its own; that its fruit, vegetables, fresh meat, etc. are imported from the coast ports, America, or Australia; second, that all dry goods come from home and are purchased at rates that will cover the buyer's loss upon exchange? To make it quite plain, five pounds' worth of goods would cost him $63.00 instead of $50.00, plus freight. Is he to be the loser of this sum? Certainly not. He therefore raises his prices to meet the emergency. So that an article costing $1 at Christmas is now charged $1.25, and by reflex action this affects the whole community; for food stuffs being dearer, labour becomes more expensive, and so on *ad lib.*; an item brought home by the fact that whereas in 1901 a ricksha puller could be engaged for $6.50 per mensem, he now justifibly demands $9.00.

### GLASGOW NURSES' CO-OPERATION.

Mr. John Stuart Bannatyne writes from Glasgow: I have to-day read the letter which appears in your issue of March 28th purporting to be from "One of the Staff," and while I do not appreciate mud-slinging, especially under cover of a *nom-de-plume*, I cannot but admire the chivalry which prompts your correspondent to defend an executive who cannot defend themselves. Your correspondent wonders what I mean by calling the nurses "servants." If she had perused the new constitution which she upholds she would there have discovered that the nurses are referred to as "servants" of the co-operation. At the annual meeting I pointed this out and stated how unfavourably situated according to Scots law servants are as regards dismissal, character, and money compared with "members," and as the Co-operation's solicitor, who was present, did not contradict me, his views doubtless coincided with mine. In face of this, will your correspondent still allege that my statements were "untrue, inaccurate, misleading, and utter nonsense?" The annual meeting *was* adjourned, but your correspondent—not of course with any intention of misleading—omits to mention that the executive strenuously opposed the adjournment. In addition the executive had, before adjournment was decided upon, refused to explain the constitution, although that was the time and place for explanations; yet some weeks later, as their own circular of January 22nd shows, they explained the constitution privately to four of the 168 nurses. Is this what your correspondent refers to as "open and above-board"? Your correspondent further alleges that I am not a member of the Co-operation. If she will refer to the secretary's books she will find that I was a member at the date of my exclusion from the meeting which adopted the constitution. That constitution she admires despite the fact that it contains, amongst other privileges (?), power to "solicit and collect" money for the upkeep of the home, and thus converts a self-supporting institution into a charitable organisation. British people generally desire to subsist upon their own earnings rather than to benefit by the proceeds of beggary, and as "One of the Staff" seems an exception to the rule, your readers will scarcely be disposed to credit her remarks. Though your correspondent claims to possess a complete knowledge of the workings of the executive she has also overlooked the written apology which has already been referred to in your columns. I now call upon her to disclose in her reply the terms of that apology and whether it was made voluntarily.

### PROPOSED "NATIONAL TRAINING SCHOOL FOR MIDWIVES."

Miss Alice S. Gregory writes from The Deanery, St. Paul's: Allow me to express my fullest concurrence in your opinion that a three years' training in general nursing would be better for a midwife than one lasting only for 18 months. Unfortunately, however, our three years' nurses have shown no inclination to step into the breach. I appeal to the matrons of our hospitals and infirmaries to say what proportion of their fully-trained nurses have embraced, or are proposing to embrace district midwifery as a life work. Also what relation the numbers would bear to the 60 or 70 per cent. of confinements throughout England, which we are removing from the hands of the untrained midwives, and for which we are thereby rendering ourselves responsible. And yet, in spite of the acknowledged reluctance of nurses to adopt this monotonous though extremely responsible calling, it is work that must be done, and the maternity patients need always some measure of nursing skill, varying according to the complexity of the case. We have all met with "nurses," who on investigation have served for a period of six or twelve months in some hospital, and being responsible to no particular organisation, are not too conscientious in their method of obtaining work. It would, indeed, be deplorable to add to their number, but it is obvious that this risk is incurred by all the many hospitals who consent to take paying probationers for short periods. In one case the danger will be minimised by the obvious advantage to the midwives of being kept on the register of a central organisation, which can only be the case as long as they conform to its rules. Their certificates will refer to midwifery and monthly nursing only, and should they therefore elect to join the ranks of semi-trained general nurses, they will lose the benefit of their two years' work, and reduce themselves to the level of those who, having served some hospital for three months, offer themselves to the public as nurses, on the sole guarantee of their own word. It is as advocates for a two years' training for midwives—versus three months' without instruction or experience in nursing—that we stand before the public. When this point, with all its necessarily entailed expenses, is conceded, I think it will be time enough to discuss whether three years' were not better. Russia is at present the only European country that insists on so prolonged a course, and this only for its country midwives. A two years' training is obligatory in France, Belgium, Holland, and, I believe, Italy and Sweden.

### Appointments.

[No charge is made for announcements under this head, and we are always glad to receive, and publish, appointments. But it is essential that in all cases the school of training should be given.]

ASTON MANOR DISTRICT NURSING ASSOCIATION.—Miss Alice Price has been appointed lady superintendent. She was trained at the Spring Hill Infirmary, Birmingham, after which she did private nursing in Paris. For the last seven years she has been on the staff of the Birmingham District Nursing Society, which is affiliated to the Queen's Jubilee Nursing Institute.

DURHAM COUNTY HOSPITAL.—Miss Emily Mann has been appointed senior sister. She was trained at Durham County Hospital, and has since been staff nurse at the Memorial Hospital, Kendal, and sister of the children's ward at West Ham Hospital.

EYE AND EAR INFIRMARY, LIVERPOOL.—Miss F. May Robinson has been appointed sister. She was trained at Southport New Infirmary, and has since been staff nurse and night charge nurse at the Warrington Hospital.

GENERAL HOSPITAL, ST. JOHN'S, NEWFOUNDLAND.—Miss Southcott has been appointed nursing superintendent. She was trained at the London Hospital, and for midwifery at the Plaistow Maternity Home.

NURSING HOME, IPSWICH.—Miss A. Mattinson has been appointed district nurse. She was trained at the Manchester Royal Infirmary, subsequently becoming staff nurse. She has since been nurse at the Home for Epileptics, Maghull, Liverpool, and charge nurse at the Carmarthenshire Infirmary.

PADDINGTON GREEN CHILDREN'S HOSPITAL. — Miss Margaret Fitch has been appointed home sister. She was trained at the County Hospital, Ryde, and has since been charge nurse at the Brook Hospital, Woolwich, staff nurse at the London Temperance Hospital, sister at the National Hospital for the Paralysed and Epileptic, Queen's Square, London, and night sister at the City of London Hospital for Diseases of the Chest, Victoria Park, E.

PRESTWICH UNION INFIRMARY.—Miss Catherine Woodward has been appointed superintendent nurse. She was trained at the Birmingham Infirmary, and has since been nurse at the Union Infirmary, Hoole, Chester.

### The Nurses' Bookshelf.

BURDETT'S OFFICIAL NURSING DIRECTORY, 1903. Containing an Outline of the Principal Laws affecting Nurses, a Classified List of Nursing Institutions and Private Nursing Branches of Hospitals in the United Kingdom and Abroad, and a Directory of Nurses. Compiled and Edited, with the assistance of a small committee of Medical Men and Matrons, by Sir HENRY BURDETT, K.C.B. (London: The Scientific Press, Limited, 28 & 29 Southampton Street, Strand. Entered Stationers' Hall. Price 3s. net.)

THIS useful directory, which has now reached its fifth year of life, carries its own recommendation in the heading above. Doctors, nurses, and private individuals also will find the supplementary chapters of great service in their search for suitable and reliable nursing institutions in Great Britain and abroad. Its small price and size brings it within reach of the purses and pockets of nurses, containing as it does so much information which they would otherwise have to seek in more expensive, bulkier volumes. Such a directory should find a place in all public libraries or reading-rooms for the use of strangers and others who have no means of discovering the local nursing institutions, while the Post Offices and public telephone call offices would be rendering the public a great service by setting an example of providing copies for reference. Though every pains is taken by the editor and his staff to render the volume as reliable as possible with regard to the address, status, etc., of every nurse and institution whose name is recorded, perfect accuracy can only be ensured by the hearty and prompt cooperation of the nurses themselves and the matrons of institutions. We would therefore endorse the prefatory note by strongly impressing on nurses the importance both to themselves and the public, of at once communicating to the editor any change of address, occupation or status, and of filling up accurately and posting by return, the forms sent yearly for revision up to date. An asterisk against the names of those who have not replied to the yearly note of revision has been adopted, so that the editorial staff is not responsible for the accuracy of such entries. We regret to note so many of these asterisks, and trust that the next issue will be as nearly as possible quite free of them, and be thereby enabled to fulfil its most useful destiny without let or hindrance.

THE ENGLISHWOMAN'S YEAR BOOK AND DIRECTORY. 1903. (London: Adam and Charles Black.)

THIS useful volume now makes its appearance for the twenty-third time, and is thoroughly revised up to date. Education, science, medicine, public work, sports and pastimes have all been treated by those who know their subjects well, whilst a new article has been contributed on pharmacy for women, and another on house decoration. There is also a list of books published during the past year, and a summary of the events of the year, chiefly as they concern women. The editor in her preface expresses a hope that all readers who may have fuller information on any subject treated within the cover of the "Englishwoman's Year Book" than is therein contained, will kindly communicate with her and thus help to augment the value of the next edition.

### "The Hospital" Convalescent Fund.

THE Hon. Sec. begs to acknowledge with many thanks the receipt of 2s. 6d. and 6s. from the Travel Editor of THE HOSPITAL.

### Echoes from the Outside World.

### The King's Visit to Portugal.

The King reached Lisbon on Thursday of last week. As soon as the yacht came to anchor in the Tagus, the Portuguese royal barge, built in the eighteenth century, rowed by 80 of the royal bargemen in picturesque red shirts and antiquated red and gold bonnets, shot out of the Arsenal Basin conveying King Charles on board the *Victoria and Albert*. A great cheer went up as the Kings stepped ashore, both wearing Admiral's uniform, that of King Charles crossed by the blue ribbon of the Garter, and that of King Edward by the rainbow ribbon of his Portuguese order. The royal host and the royal guest, amidst the plaudits of the multitude, drove together along the streets to the Necessidades Palace in a wonderful gilded and emblazoned coach, 200 years old, drawn by four pairs of horses. Another coach, occupied by members of the suite, was a present from Louis XIV., a third given by a Pope in the eighteenth century, and yet a fourth, a gift years before from the Austrian Emperor, all marvellously gorgeous. Friday was devoted to an excursion to the historic castle of Cintra, a site of exceptional loveliness. The Kings drove in a carriage drawn by four splendid mules. Amongst the triumphal arches erected was one bearing the words "God save the King," and the road was very gay, lined by peasants in their picturesque national costumes. On Saturday the two Kings spent the morning at the world-renowned Geographical Society, and the ladies in the balconies poured cornucopias of rose leaves upon their Majesties' heads. This kind attention much amused King Edward. In the afternoon, in company of the Queen-mother, Maria Pia, he attended a pigeon-shooting competition, and watched the shooting for King Edward's Cup, for which King Charles unsuccessfully competed. In the evening there was a gala performance at the São Carlos Theatre. On Sunday morning King Edward attended service in the Anglican Church, and in the evening he was entertained at a State banquet in the Palace. On Monday he visited the Convent of the Dominican Monks at Bom Successio.

### Movements of Royalty.

King Edward has made arrangements to visit Italy towards the end of the present month, hoping to arrive at Naples about the 25th inst. on board the *Victoria and Albert*. He will repair to Rome, where the whole of the Italian Royal Family will be present to meet him. He will remain four or five days, and will afterwards go on to Paris, where he will hold official intercourse with the President of the French Republic. The French and Italian papers are enthusiastic in their rejoicings over the coming events. The German Emperor has been a guest of the Danish King the same time as Queen Alexandra. There was a gala dinner in the King's apartments in the palace the night of his arrival, when the German Emperor escorted Queen Alexandra to the table and the King the Dowager-Empress of Russia. On Friday the German Emperor inspected a china factory and had a long drive during the day, but attended a reception in the evening given to all the Royal guests by the Crown Prince and Princess of Denmark. The hand of Queen Alexandra is traced by her subjects in the order which has been issued by the Lord Chamberlain as to the Court to be held at the Palace of Holyrood on Tuesday, May 12th. Ladies who receive an invitation, as well as those who are presented, many of whom will be the daughters of ladies presented at Drawing-rooms held during the reign of her late Majesty, Queen Victoria, are all to wear morning dress, with bonnets. The term "bonnet," the notice explains, may be taken to mean either bonnets or toques, but not hats.

### The new Dean of Canterbury.

The successor of Dr. Farrar as Dean of Canterbury is the Rev. Henry Wace, D.D., and the Prime Minister in recommending his appointment to the King has thus again given effect to his policy of nominating scholars to Deaneries. Dr. Wace, who is 66 years of age, is a native of London, and distinguished himself at Brasenose College, Oxford. His first curacy was that of the poor parish of St. Luke's, Berwick Street, Soho, and his second that of St. James's, Piccadilly. From 1872, however, he did not hold any parochial appointment until 1896. He has been in succession chaplain of Lincoln's Inn, Professor of Church History at King's College, Principal of King's, and Preacher of Lincoln's Inn. In 1896 he resigned both the preachership at Lincoln's Inn and the principalship of King's College, in order to become rector of St. Michael's, Cornhill. He is a man of great literary ability and a profound thinker, whose intellectual gifts have now received no more than just recognition.

### The Oldest Englishwoman.

Many will regret to hear of the death of Mrs. Margaret Neave, the well-known centenarian, for the fame of her longevity had spread far beyond her place of residence. She was born at Guernsey in 1792, and would have been 111 on her next birthday, but her health began to fail in November, and she died of senile decay last week on her charming estate at St. Peter Port, where she had lived as a widow for more than fifty years. In her younger days she had travelled much, having been all over Europe except to Portugal, and spoke several languages well. When she was 81 she paid a second visit to Poland. As recently as 1895 she used to walk a mile and a-half to church every Sunday on the arm of her nephew, and until just after her 101st birthday she could be seen almost daily in the summer weeding in front of her residence, Rouge Huyshe. As a child she fell over the banisters, and was unconscious for some days, but otherwise seldom suffered ache or pain. She was sent to Bristol to school at 10 years old, and was 13 at the time of the battle of Trafalgar and could quite well remember the event. She had conversed with several of Napoleon's Generals, Marshal Ney amongst them, and was always very proud of a belt-buckle worn by one of the Imperial Guard, which she picked up herself on the field of Waterloo after the battle.

### Anti-Cigarette Crusade in Canada.

For twenty years the women reformers of Canada have been steadily waging an anti-cigarette war. One great desire was to procure legislation if possible to prevent the boys and girls of the Dominion smoking away their brains and their health. Physicians state that young people of both sexes from eight years of age are often thoroughly inured to the habit, and at one of the reformatory schools 75 per cent. of the boys under 14 were found, when admitted, to be inveterate smokers. In the public day schools it is reported that much smoking of cigarettes in secret by the girls goes on, and either the parents fail to discover the contraction of the habit till too late to stop it, or they do not care to interfere. In every province of Canada except Quebec there is legislation against the sale of cigarettes to boys and girls under 16 or 18, but nowhere is the law enforced. Now, however, the reformers believe that they have at last made a step forward. The Dominion House of Commons last week adopted a motion in favour of prohibiting the import, manufacture, and sale of cigarettes, and it will be followed by the introduction of a Government Bill by the Minister of Justice embodying the terms of the resolution.

### For Reading to the Sick.

EASTERTIDE.

Alleluia! Alleluia! Hearts to Heav'n and voices raise,
Sing to God a hymn of gladness, sing to God a hymn of praise;
He, who on the cross a victim for the world's salvation bled,
Jesus Christ, the King of Glory, now is risen from the dead.

Christ is risen, Christ the first fruits of the Holy harvest-field,
Which will all its full abundance at His second coming yield;
Then the golden ears of harvest will their heads before Him wave,
Ripened by His glorious sunshine, from the furrows of the grave.

Christ is risen, we are risen; shed upon us heavenly grace,
Rain and dew, and gleams of glory from the brightness of His face,
That we, with our hearts in Heaven, here on earth may fruitful be,
And by angel hands be gathered, and be ever, Lord, with Thee.
*Bishop C. Wordsworth.*

The love of the dying Christ, the joy of the risen Christ, the sympathy of the ascended Christ, and the glory of the returning Christ be with you and comfort your hearts.
*Anon.*

I am the Resurrection and the Life; he that believeth on Me though he die yet shall he live; and whosoever liveth and believeth on me shall never die. Christ, in a word, is the Resurrection of the dead; Christ is the life of the living: He is the Resurrection because He is the Life. Death is the dream, the shadow; and not life, as we hastily judge, who measure being by our senses.

This, then, is the first point. Life, eternal life, and, therefore, the Resurrection as included in it, is brought to men by Christ now. Christ shows by His word, and by the speaking fact of the recall of Lazarus to earthly life, that what we see is but a small part of what we are; that physical death touches only the circumstances of our present existence; that dissolution is the condition of a new form of life, but not an interruption, still less the close of life.

By every detail of this history we are encouraged to look below the surface of things, to realise how life, true life, triumphs over death even through death; to regard the restoration of Lazarus not as a mere marvel only, but as a type of the constant action of God, Who preserves through every vicissitude all that which makes us what we are; to know by that staying of the power of corruption, by that call to renewed activity, that Christ as He is the Food to support us, the Light to guide us, is also the Life—infinite and eternal—by which we live.

If we believe in a living Christ, the Son of God, that faith contains treasures of wisdom which later experience will teach us to make our own. The years as they pass may leave us a sad inheritance of weakness and death; but in due time Christ will reveal Himself to us, even here, in this chequered scene of loss and conflict, as the Resurrection and the Life, the Life whereby He quickens us for new labour, the Resurrection whereby He gives back to us the past transfigured for nobler uses.—*Bishop Westcott.*

### Notes and Queries.

**The Editor is always willing to answer in this column, without any fee, all reasonable questions, as soon as possible.**

**But the following rules must be carefully observed:—**

1. **Every communication must be accompanied by the name and address of the writer.**
2. **The question must always bear upon nursing, directly or indirectly.**

**If an answer is required by letter a fee of half-a-crown must be enclosed with the note containing the inquiry, and we cannot undertake to forward letters addressed to correspondents making inquiries. It is therefore requested that our readers will not enclose either a stamp or a stamped envelope.**

*The Midwives Act.*

(8) Can you tell me, please, when the new Midwives Act came in force, and how can cottage women not at present qualified get their certificates?—*G. H.*

The Act is already in operation, and as to the second point you had better write to the Secretary, Midwives' Club, 12 Buckingham Street Strand, W.C.

*Probationers of* 17 *and* 18.

(9) 1. Is there any position in a hospital which intending probationers can occupy till old enough for training? 2. Or can they go into a children's hospital so young? 3. Will a provincial hospital's preliminary training increase their difficulty in getting into a London hospital?—*No.* 37.

1. There are no positions likely to be given to such young girls. 2. None of the larger children's hospitals in London accept probationers under 20; but you might inquire at the Alexandra Hospital for Children with Hip Diseases, Queen Square, Bloomsbury, W.C.

*Home for Convalescent Infectious Patients.*

(10) Can you tell me how to make known my private home near Bristol for patients after infectious diseases? Terms very moderate. *Sister.*

You can only advertise and try to get yourself known amongst doctors; or you might inform the authorities of fever hospitals where private patients are taken.

*Probationer in Children's Hospital.*

(11) I wish to train in a children's hospital. Would it be any use my writing to the Children's Hospital in Great Ormond Street? Must I pay a premium? I am 24.—*M. L.*

By all means write to the hospital you name, but they have a great many applications, and you may have to wait. You will not have any premium to pay.

*Training.*

(12) I should be glad if you will tell me where I ought to apply, as I am anxious to be trained as a nurse in Glasgow.—*E. A.*

There are several training schools for nurses at Glasgow (see Burdett's "Nursing Directory"), the largest being the Glasgow Royal Infirmary.

*Catholic Nursing Homes.*

(13) Will you kindly let me know if there are any nursing associations and homes for nurses who are Catholics?—*M. A.*

Nursing institutions are generally non-denominational. There are, however, private homes and guilds for nurses who are Roman Catholics. You had better advertise.

*Akouphone.*

(14) Can you help me in any way in finding out details of an American invention for enabling the deaf to hear. I am very much interested in a boy of 12 who became deaf shortly after birth, and I hear that by applying this instrument—the akouphone—to the ear, the hearing of the deaf can be restored. I believe that there is no English ageny, and would like to know if the instrument is expensive.—*Stalrock.*

The address is the Akouphone Company, 42–48 East 20th Street, New York. There is no agency in England, and the instrument is expensive. You had better consult a medical man on the point.

**Standard Nursing Manuals.**

"The Nursing Profession: How and Where to Train." 2s. net; 2s. 4d. post free.
"Nursing: Its Theory and Practice." (Revised Edition). 3s. 6d. post free.
"Surgical Ward Work and Nursing." (Revised Edition). 3s. 6d. net.; 3s. 10s. post free.
"Practical Handbook of Midwifery." (New Edition). 6s. net; 6s. 3d. post free.
"Notes on Pharmacy and Dispensing for Nurses." 1s. post free.
"Fevers and Infectious Diseases." 1s. post free.
"The Art of Massage." (New Edition). 6s. post free.

### Travel Notes.

By Our Travelling Correspondent.

### CXIX.—ON THE SPANISH FRONTIER.

Three different correspondents have asked for information concerning a holiday to be spent in this neighbourhood, and I have therefore decided to write a few articles dealing with the subject, for it is impossible to enter fully into the matter in our correspondence column.

### A Short Spanish Holiday is Expensive.

Living in Spain (provided you stay some time) is cheap, because the rate of exchange is very favourable to us; but the journey is dear because it is a long distance, and hotels are not so reasonable as in France or Italy; therefore if economy is an object I should say do not go far into the country unless you have two or three months at your disposal.

I should rather recommend a residence at St. Jean-de-Luz, the frontier town on the French side, and from there to make expeditions into Spain. St. Sebastian is easily reached, also Fontarabia and Pasajes, and a trip to Burgos is not difficult. I spent six happy months in St. Jean-de-Luz, and I think I may claim that I know every mile of the country all round.

### Expenses of the Journey.

Second class return will be (viâ Newhaven and Dieppe) £6 10s., and if you can travel straight through without a break you need not incur further expense, because a well-stocked tea-basket will hold all the food you need. If you have not more than a month or six weeks to spend, I should advise an hotel; that of La Poste will entertain you royally for 7f. per day, under £2 per week; but if you are remaining two or three months a furnished apartment is the thing. We occupied one of two sitting-rooms, three bedrooms, and a kitchen at the modest price of 10f. per week. A servant costs 25f. per month and living is very cheap; but for a short time this arrangement is not worth while, nor does it answer well unless you speak French fluently. It is not that you will be cheated—far from that, the people are as honest as the day—but there naturally arise many occasions when knowledge of the language is an absolute necessity in rooms of your own. St. Jean is a dear little town, very southern in appearance, with whitewashed houses overhanging green, pink, and chocolate balconies and narrow streets. The church, a really magnificent structure in its own particular style, is called the Basque Cathedral, and in it you recognise some of the peculiar national characteristics of the strange and mysterious race who mingle, without mixing, with other people. Their language remains distinct, and very many of the older men and women cannot speak French at all. My servant, a handsome widow of 35, could speak French certainly after a fashion—just so that I could understand her—but the moment she was off the beaten track in conversation she was lost. The church resembles a vast hall without pillars, very dim and sombre; on a raised platform at the east end is the high altar. All this part is heavily gilt, as is the Spanish mode, and richly painted in dark mellow shades. It is not the custom for the Basque men and women to sit together, so the walls are furnished with three shallow galleries in carved black oak in which the men sit, whilst the fairer sex occupy the floor. Their appearance is singular, for it is correct on Sundays for the women of the middle class to wear a distinctive cloak—alas! fast disappearing. It is black, round, and vast, gathered into a hood which comes far over the face, and is garnished with a deep fall of black Spanish lace almost totally concealing the face of the wearer. The poorer women wear nothing on their heads except a tiny silk handkerchief twisted over the knot of hair at the back.

### The Possessive Prayer Carpet.

A very singular custom prevails here of appropriating a portion of the church floor by laying down a small black carpet. It varies in size, and the owner's chair is planted on it, proclaiming to the initiated that the worshipper's relatives are interred beneath or close outside the walls. It is considered a great breach of decorum to plant your kneeling-chair on one of these carpets without first asking permission.

In this grand old church Louis XIV. was married to Maria Theresa, and if you can make friends with Monsieur l'Abbé he will show you the stately vestments that were used on that occasion. He is a most courteous and charming gentleman, of whom I retain the pleasantest recollection. In a time of great anxiety and difficulty he was our kindest friend and helper.

The night before the wedding Louis slept in a fine arcaded house in the Place, now called the Café Suisse. It has turrets at the corners, so that you will easily recognise it; and the bride-elect occupied a curious old house behind the fish quay, where the sardine boats unload. In the comfortable Hôtel de France, alas! now given up, I slept beneath a regal quilt said to have been used by Louis XIV., and I can well believe the legend. The work is certainly of that period. The groundwork is stout unbleached linen, and on it, parrots of unknown development and strange tint, promenade majestically round the border.

### Mountain Excursions.

There are two mountains easily negotiated from St. Jean-de-Luz—La Rhune and the "Trois Couronnes."

To ascend La Rhune (most interesting because it was the basis of so many military operations of Wellington in 1813) it is best to start from Ascain. This little village you reach from St. Jean by omnibus (or if you are cyclists the matter is still easier), and return by Sare, from whence also an omnibus returns to St. Jean; hours, however, do not always fit, and if you are a party of three or four it comes nearly or quite as cheap to take a carriage. It is quite an easy ascent, and if you are not good walkers mules are always ready for you either at Ascain or Sare. "Trois Consonnes" is equally easy as far as personal exertion is concerned, but not quite so attainable because it is further off. I have not climbed it myself, but my friends did; and though there is a fine view they did not think the expedition so enjoyable as that to La Rhune.

---

Rules in Regard to Correspondence for this Section.—All questioners must use a pseudonym for publication, but the communication must also bear the writer's own name and address as well, which will be regarded as confidential. All such communications to be addressed "Travel Editor, 'Nursing Section of *The Hospital*,' 28 Southampton Street, Strand." No charge will be made for inserting and answering questions in the inquiry column, and all will be answered in rotation as space permits. If an answer by letter is required, a stamped and addressed envelope must be enclosed, together with 2s. 6d., which fee will be devoted to the objects of "The Hospital" Convalescent Fund. Any inquiries reaching the office after Monday cannot be answered in "The Hospital" of the current week.

